# Pharmacogenetic Risk Stratification in Angiotensin-Converting Enzyme Inhibitor-Treated Patients with Congestive Heart Failure: A Retrospective Cohort Study

**DOI:** 10.1371/journal.pone.0144195

**Published:** 2015-12-03

**Authors:** Karl Emil Nelveg-Kristensen, Majbritt Busk Madsen, Christian Torp-Pedersen, Lars Køber, Martin Egfjord, Henrik Berg Rasmussen, Peter Riis Hansen

**Affiliations:** 1 Department of Cardiology, Gentofte University Hospital, Hellerup, Denmark; 2 Institute of Biological Psychiatry, Mental Health Centre Sct. Hans, Copenhagen University Hospital, Roskilde, Denmark; 3 Institute of Health, Science and Technology, Aalborg University, Aalborg, Denmark; 4 The Heart Centre, Rigshospitalet, Copenhagen University Hospital, Copenhagen, Denmark; 5 Department of Nephrology, Rigshospitalet, Copenhagen University Hospital, Copenhagen, Denmark; Children's National Medical Center, Washington, UNITED STATES

## Abstract

**Background:**

Evidence for pharmacogenetic risk stratification of angiotensin-converting enzyme inhibitor (ACEI) treatment is limited. Therefore, in a cohort of ACEI-treated patients with congestive heart failure (CHF), we investigated the predictive value of two pharmacogenetic scores that previously were found to predict ACEI efficacy in patients with ischemic heart disease and hypertension, respectively. Score A combined single nucleotide polymorphisms (SNPs) of the angiotensin II receptor type 1 gene (rs275651 and rs5182) and the bradykinin receptor B1 gene (rs12050217). Score B combined SNPs of the angiotensin-converting enzyme gene (rs4343) and ABO blood group genes (rs495828 and rs8176746).

**Methods:**

Danish patients with CHF enrolled in the previously reported Echocardiography and Heart Outcome Study were included. Subjects were genotyped and categorized according to pharmacogenetic scores A and B of ≤1, 2 and ≥3 each, and followed for up to 10 years. Difference in cumulative incidences of cardiovascular death and all-cause death were assessed by the cumulative incidence estimator. Survival was modeled by Cox proportional hazard analyses.

**Results:**

We included 667 patients, of whom 80% were treated with ACEIs. Differences in cumulative incidences of cardiovascular death (P = 0.346 and P = 0.486) and all-cause death (P = 0.515 and P = 0.486) were not significant for score A and B, respectively. There was no difference in risk of cardiovascular death or all-cause death between subjects with score A ≤1 vs. 2 (HR 1.03 [95% CI 0.79–1.34] and HR 1.11 [95% CI 0.88–1.42]), score A ≤1 vs. ≥3 (HR 0.80 [95% CI 0.59–1.08] and HR 0.91 [95% CI 0.70–1.20]), score B ≤1 vs. 2 (HR 1.02 [95% CI 0.78–1.32] and HR 0.98 [95% CI 0.77–1.24]), and score B ≤1 vs. ≥3 (HR 1.03 [95% CI 0.75–1.41] and HR 1.05 [95% CI 0.79–1.40]), respectively.

**Conclusions:**

We found no association between either of the analyzed pharmacogenetic scores and fatal outcomes in ACEI-treated patients with CHF.

## Introduction

The prevalence of congestive heart failure (CHF) is increasing and CHF now affects approximately 10% of subjects above 60 years, with an overall 5-year mortality rate remaining at 50% [1–3]. Angiotensin-converting enzyme inhibitors (ACEIs) reduce mortality and morbidity in patients with CHF and therefore represent a cornerstone in the current pharmacologic management of this disease [4, 5]. The clinical response to treatment with ACEIs, however, displays substantial inter-individual variability in terms of both safety and efficacy, and genetic variability of patients contributes to this phenomenon [6–8]. Accordingly, selected genetic variants have been proposed for pharmacogenetic risk stratification of patients treated with ACEIs in attempt to individually tailor such treatment [9–11]. Notably, two distinct genetic scores based on single nucleotide polymorphisms (SNPs) in the angiotensin II receptor type 1 gene (*AGTR1*) and bradykinin receptor B1 gene (*BDKRB1*), as well as the ACE gene (*ACE*) and ABO blood group genes (*ABO*), respectively, were recently found to predict ACEI efficacy and ACE activity in European patients with stable ischemic heart disease (IHD) and in Asian patients with young-onset hypertension, respectively [9–12]. Therefore, we examined the predictive value of these two genetic scores in a cohort of ACEI-treated patients with CHF.

## Materials and Methods

### Study population and endpoints

This retrospective cohort study was based on patients participating in the previously reported Echocardiography and Heart Outcome study (ECHOS), a randomized, double-blinded, placebo-controlled trial of nolomirole (a mild inhibitor of the sympathetic nerve system) in patients with severe CHF, where nolomirole was found to possess no beneficial or harmful effects after 2.5 years of follow-up [13]. In the period 2001–2002, ECHOS included 1000 patients >18 years of age from centers in Denmark, Norway and Sweden admitted with New York Heart Association (NYHA) class II-IV CHF and an echocardiographic left ventricular wall motion score index ≤1.2 (corresponding to an ejection fraction ≤ 35%) [13]. Among exclusion criteria were uncorrected hemodynamically significant valvular disease and acute myocardial infarction within one month. Approximately 80% of ECHOS patients were treated with ACEIs and for the present study, criteria for inclusion were participation in ECHOS, Danish citizenship and availability of DNA for genotyping. Patients were followed from hospital discharge until occurrence of a study endpoint or a maximum of 10 years. Our primary endpoint was cardiovascular (CV) death and the secondary endpoint was all-cause death. Since nolomirole had no effects on clinical outcomes in the ECHOS trial and as the drug therefore was discontinued hereafter, we did not differentiate between patients who received nolomirole and those who received placebo. Information on baseline treatment with ACEIs, beta-blockers, aldosterone antagonists, diuretics, and insulin was available from the ECHOS dataset, which also supplied information on NYHA class, wall motion score index, IHD, diabetes mellitus, chronic obstructive pulmonary disease, atrial fibrillation, body mass index, and smoking status. Information on vital status and individual average annual gross income (socioeconomic status) throughout the 5-year period prior to inclusion was obtained from the National Causes of Death Registry and the Danish Central Person Registry, respectively [14].

### Genetic analyses

The first genetic score (score A) used for the current study was previously found to be associated with reduced ACEI treatment benefit in European patients with stable IHD, and was based on three SNPs, i.e., rs275651 and rs5182 located in *AGTR1* and rs12050217 in *BDKRB1*, respectively [11]. The second genetic score (score B) was previously reported to be associated with systemic ACE activity and response to ACEI treatment in patients of Asian descent with young-onset hypertension [10]. This score also consisted of three SNPs, i.e., rs4343 located in *ACE*, which is in high linkage disequilibrium (LD) with the *ACE* I/D polymorphism (rs1799752), and rs495828 and rs8176746 of *ABO*, respectively. In the original reports, both genetic scores A and B assigned one score point to the respective responder allele of each genotype [10, 11]. Because alleles can occur in a homozygous or heterozygous form, each genotype could contribute with a maximum of two score points. As an exception to this rule, however, score B assigned only 1 point for rs495828 if the responder allele occurred in homozygote form, and 1 point for rs8176746, irrespective of whether the responder allele appeared in heterozygous or homozygous form [10]. Thus, there were a maximum of 6 achievable points with score A, but only 4 achievable points for score B. Moreover, due to the relatively undersized sample in the current study, we condensed both genetic scores to include only three individual score levels which was also done in the original study for score A, i.e., with ≤1, 2, and ≥3 score points [11]. By this method, the number of patients within each score level was increased, as was the statistical power of the study. Because recent fine-mapping of *ACE* revealed a break point in an LD block that was significantly associated with ACE activity in an Asian population, we examined an additional SNP in *ACE* (rs4353) in order to tag the gene on either side of the recombinant break point [12]. The seven selected SNPs were genotyped by use of TaqMan SNP genotyping reagents, specific primers and probes (Applied Biosystems, Foster City, CA, USA). We used two different PCR master mixtures. For the genotyping of rs495828, rs5182, and rs275651, the TaqMan Universal PCR Master Mix was used (Applied Biosystems, Foster City, CA, USA). Genotyping of SNPs rs4343, rs4353, rs8176746 and rs12050217 was done using KAPA PROBE FAST qPCR Kit Master Mix (2x) ABI Prism (Kapa Biosystems, Inc., Wilmington, MA, USA). Amplification conditions were as recommended by the manufacturers of the PCR master mixtures.

### Statistics

Chi-square tests were used to examine if the genotype frequencies were distributed according to the Hardy-Weinberg equilibrium. Information on six additional *ACE* SNP genotypes in European and Asian (Han Chinese) populations, respectively, were obtained from the International HapMap Project [15]. Haploview was applied to determine and visualize LD relationships between SNPs in this gene [16].

Incidence rates (IRs) for each of the two study endpoints expressed as events per 100 person-years were calculated for patients with ≤1, 2 or ≥3 score points for score A and B, respectively. The Chi-Square test and Student’s t-test were used to examine differences in categorical and continuous baseline characteristics. Cumulative incidences were assessed by Gray’s nonparametric test as well as the Aalen-Johansen cumulative incidence estimator, both modeled for compering risk with CV death [17]. Cox proportional hazard analyses were used to model survival. In analyses of survival data, patients entered the model at the date of discharge from the index hospitalization where they were randomized in the ECHOS study. The survival analyses were performed at up to 10 years of follow-up (main analysis) and 5 years of follow-up (sensitivity analysis), respectively. The analyses exclusively included ACEI-treated patients, and patients without ACEI treatment were only included for selected additional analyses to examine the predictive value of the genetic scores in non-ACEI-treated patients, as well as for an over-all assessment of interactions between the genetic scores and ACEI treatment status. Additionally, the predictive capacity of score A was evaluated separately in ACEI-treated patients with ischemic and non-ischemic CHF, respectively, as the value of this score was originally demonstrated in patients with IHD [11]. Finally, subgroup analyses of CV death in patients treated with the three most frequently used ACEIs in the study were done for both scores. Survival analyses were adjusted for age, sex, IHD, NYHA class, wall motion score index, diabetes mellitus, chronic obstructive pulmonary disease, and insulin treatment, which have all previously been associated with mortality in the ECHOS population [18]. In addition, analyses were adjusted for socioeconomic status. The proportional hazard assumption, linearity of continuous variables, and absence of interaction, respectively, were tested and found valid if not otherwise stated. A two sided P value ≤0.05 was considered statistical significant. Analyses and data management were performed in SAS version 9.3 (SAS Institute Inc. Cary, North Carolina) and R version 3.0.2 (R Foundation for Statistical Computing, Vienna, Austria).

### Ethics

This project was approved by the Danish National Committee on Health Research Ethics (protocol no. H-15006089) and by the Danish Data Protection Agency (protocol no. 2007-58-0015/GEH-2010-001). All files were anonymized prior to data management and statistical analyses. ECHOS was approved by authorities in the participating countries as well as by relevant ethics committees and was conducted in accordance with the Declaration of Helsinki III and Guidelines for Good Clinical Practice in the European Union. All patients gave their written informed consent prior to participation [13].

## Results

### Study population

Of the 1000 patients originally randomized in ECHOS, 667 patients of Danish citizenship had DNA available for genotyping. Of these 525 (79%) patients were treated with ACEIs and 349 (52%) patients had IHD. The three most frequently used ACEIs were trandolapril (40% of patients), ramipril (25%), and perindopril (16%), which were dispensed in mean daily doses of 2.4 (SD 1.2) mg, 6.2 (SD 3.1) mg, and 3.3 (SD 1.0) mg, respectively (Tables 1 and 2). There were no significant differences in baseline characteristics between patients with different number (≤1, 2, or ≥3) of score points for scores A and B, except for the distribution of patients treated with trandolapril in score B (Tables 1 and 2). The distribution of patients with ≤1, 2 or ≥3 score points were 293 (43.9%), 215 (32.2%) and 159 (23.8%) for score A, and 276 (41.4%), 261 (39.1%) and 130 (19.5%) for score B. The mean follow-up time for the total study population was 5.1 (SD 3.6) years. Mean (SD) follow-up times for patients with ≤1, 2 or ≥3 score points were 5.6 (3.6), 5.0 (3.6), and 5.9 (3.5) years for score A, and 5.5 (3.7), 5.5 (3.7), and 5.3 (3.2) years for score B, respectively.

**Table 1:** Baseline clinical characteristics for patients with genetic score A.

	Score A ≤1	Score A = 2	Score A ≤3	Total	P value
**Number of patients**	293	215	159	667	
**Male gender**	206 (70.3)	161 (74.9)	120 (75.5)	487	0.3756
**Age** (mean [SD] years)	70.07 (12.21)	69.91 (11.33)	70.04 (10.72)	70.01 (11.57)	0.9612
**Socioeconomic class**					
Low	169 (57.7)	140 (65.1)	98 (61.6)	407	0.2326
High	124 (42.3)	75 (34.9)	61 (38.4)	260	0.2326
**Comorbidity**					
Diabetes	48 (16.4)	35 (16.4)	30 (18.9)	113	0.7704
Ischemic heart disease	159 (54.3)	112 (52.3)	78 (49.1)	349	0.5706
COPD	59 (20.1)	46 (21.5)	38 (24.1)	143	0.6276
Atrial fibrillation	91 (31.1)	67 (31.8)	55 (34.6)	213	0.7372
**Previous coronary procedure**					
CABG	43 (14.7)	33 (15.4)	25 (15.7)	101	0.9494
PCI	19 (6.5)	11 (5.1)	11 (6.9)	41	0.7343
**Medication**					
ACEIs *	232 (79.7)	171 (79.9)	122 (78.2)	525	0.9101
- Trandolapril	91 (31.1)	74 (34.4)	47 (29.6)	212	0.6146
- Ramipril	65 (22.2)	37 (17.2)	28 (17.6)	130	0.3058
- Perindopril	38 (13.0)	28 (13.0)	20 (12.7)	86	0.9967
- Enalapril	18 (6.1)	19 (8.8)	16 (10.1)	53	0.2726
- Captopril	7 (2.4)	6 (2.8)	4 (2.5)	17	0.9617
- Lisinopril	4 (1.4)	4 (1.9)	4 (2.5)	12	0.6665
- Quinapril	1 (0.3)	1 (0.5)	1 (0.6)	3	0.9048
- Fosinopril	1 (0.4)	0 (0.0)	0 (0.0)	1	0.5290
Beta-blockers	146 (50.2)	111 (51.9)	80 (51.3)	337	0.928
Aldosterone antagonists	155 (53.3)	129 (60.3)	91 (58.3)	375	0.2611
Diuretics	287 (98.6)	208 (97.2)	153 (98.1)	648	0.5199
Insulin	27 (9.3)	13 (6.1)	7 (4.5)	47	0.1324
Nolomirole	136 (46.2)	111 (34.3)	77 (48.4)	324	0.5714
**Clinical measures**					
NYHA	2.28 (0.64)	2.27 (0.64)	2.31 (0.59)	2.28 (0.63)	0.6826
WMI	0.84 (0.23)	0.84 (0.25)	0.85 (0.23)	0.84 (0.24)	0.5595
Body mass index	26.06 (4.75)	26.02 (5.11)	26.66 (5.36)	26.20 (5.02)	0.3112
Smoker	94 (32.8)	64 (30.0)	51 (32.5)	209	0.7963

COPD: chronic obstructive pulmonary disease; CABG: coronary artery bypass graft; PCI: percutaneous coronary intervention; ACEI: angiotensin-converting enzyme inhibitor; NYHA: New York Heart Association; WMI: wall motion index.

* A total of 11 ACEI-treated patients lacked information on the specific type of ACEI.

**Table 2:** Baseline clinical characteristics for patients with genetic score B.

	Score B ≤1	Score B = 2	Score B ≤3	Total	P value
**Number of patients**	276	261	130	667	
**Male gender**	190 (68.8)	197 (75.5)	100 (76.9)	487	0.1193
**Age** (mean [SD] years)	69.64 (11.50)	70.47 (11.55)	69.88 (11.84)	70.01 (11.57)	0.7014
**Socioeconomic class**					
Low	161 (58.3)	161 (61.7)	85 (65.4)	407	0.3815
High	115 (41.7)	100 (38.3)	45 (34.6)	260	0.3815
**Comorbidity**					
Diabetes	46 (16.7)	44 (16.9)	23 (17.7)	113	0.9706
Ischemic heart disease	155 (56.2)	129 (49.4)	65 (50.4)	349	0.2593
COPD	61 (22.1)	52 (20.0)	30 (23.3)	143	0.7257
Atrial fibrillation	92 (33.6)	78 (30.1)	43 (33.1)	213	0.6709
**Previous coronary procedure**					
CABG	46 (16.7)	35 (13.5)	20 (15.4)	101	0.5842
PCI	19 (6.9)	19 (7.3)	3 (2.3)	41	0.1248
**Medication**					
ACEIs*	230 (84.2)	198 (76.4)	97 (75.2)	525	0.0350
- Trandolapril	103 (37.3)	84 (32.2)	25 (19.2)	212	0.0011
- Ramipril	56 (20.3)	48 (18.4)	26 (20.0)	130	0.8378
- Perindopril	31 (11.2)	35 (13.4)	20 (15.4)	86	0.4898
- Enalapril	20 (7.2)	20 (7.7)	13 (10.0)	53	0.6219
- Captopril	6 (2.1)	6 (2.3)	5 (3.8)	17	0.5783
- Lisinopril	4 (1.4)	3 (1.1)	5 (3.8)	12	0.1431
- Quinapril	1 (0.4)	1 (0.4)	1 (0.8)	3	0.8322
- Fosinopril	0 (0.0)	1 (0.4)	0 (0.0)	1	0.4957
Beta-blockers	133 (48.7)	137 (52.9)	67 (51.9)	337	0.6106
Aldosterone antagonists	157 (57.5)	145 (56.0)	73 (56.6)	375	0.9384
Diuretics	271 (99.3)	252 (97.3)	125 (96.9)	648	0.1538
Insulin	21 (7.7)	17 (6.6)	9 (7.0)	47	0.8778
Nolomirole	145 (52.4)	116 (44.3)	63 (48.5)	324	0.1727
**Clinical measures**					
NYHA class	2.23 (0.63)	2.32 (0.65)	2.30 (0.58)	2.28 (0.63)	0.1778
WMI	0.84 (0.23)	0.86 (0.24)	0.83 (0.24)	0.84 (0.24)	0.9681
Body mass index	25.93 (4.69)	26.17 (5.25)	26.89 (5.26)	26.20 (5.02)	0.1265
Smoker	97 (35.7)	78 (30.5)	34 (26.4)	209	0.1465

COPD: chronic obstructive pulmonary disease; CABG: coronary artery bypass graft; PCI: percutaneous coronary intervention; ACEI: angiotensin-converting enzyme inhibitor; NYHA: New York Heart Association; WMI: wall motion index.

* A total of 11 ACEI-treated patients lacked information on the specific type of ACEI.

### Population genetics

**Fig 1:**
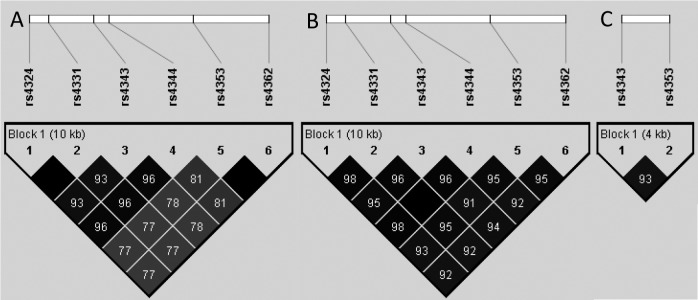
Linkage disequilibrium (LD) relationships between single nucleotide polymorphisms (SNPs) in the genes encoding the angiotensin-converting enzyme (ACE). The top of the figure shows the SNPs. A: HapMap Han Chinese population [15]; B: HapMap European population [15]; C: Echocardiography and Heart Outcome study (ECHOS) population. The strength of LD was determined by R^2^ statistics. The decrease in LD in the Han Chinese population probably reflects the previously reported recombinant breakpoint between rs4344 and rs4353 [12]. No such decrease in LD is observed for the HapMap European population and was also not observed in the ECHOS population. The *ACE* I/D polymorphism was not included in the figure as it has not been genotyped in the HapMap populations [15]. However, this polymorphism is located between rs4331 and rs4343, and is in high LD with these two SNPs [12].

Allele and genotype frequencies calculated in the total study population and in patients with and without ACEI treatment, respectively, are shown in Table 3. Furthermore, P values for the deviation from Hardy-Weinberg equilibrium are shown for each SNP in Table 3. There was a lower degree of LD in *ACE* among Han Chinese than in European HapMap individuals. This was evident from lower R^2^ values for the pairwise associations of SNP alleles across the recombinant breakpoint between rs4344 and rs4353 previously identified in Chinese subjects (Fig 1) [12]. The six examined *ACE* SNPs were highly correlated in the European HapMap population as suggested by high R^2^ values for all pairwise comparisons without evidence of a recombinant breakpoint. In the ECHOS population rs4343, a neighbour of rs4344, and rs4353 were also highly correlated (Fig 1). Hence, rs4343 and rs4353 were redundant and the latter was excluded in the subsequent statistical analyses.

**Table 3:** Allele and genotype frequencies for the total study population and patients treated with and without angiotensin-converting enzyme inhibitors (ACEIs).

	Gene	SNP	Group	MAF	Genotype frequencies			HWE P value
Score A								
	*AGTR1*							
		rs275651		T	AA	TT	AT	
			Total	0.18	0.67	0.03	0.30	0.6494
			ACEI	0.18	0.67	0.03	0.29	0.8116
			Non-ACEI	0.18	0.66	0.01	0.33	0.1460
		rs5182		C	CC	TT	CT	
			Total	0.49	0.22	0.25	0.53	0.0911
			ACEI	0.47	0.21	0.27	0.52	0.2264
			Non-ACEI	0.53	0.25	0.19	0.56	0.1403
	*BDKRB1*							
		rs12050217		G	AA	GG	AG	
			Total	0.22	0.62	0.05	0.33	0.6541
			ACEI	0.22	0.62	0.06	0.33	0.3612
			Non-ACEI	0.20	0.63	0.03	0.34	0.3786
Score B								
	*ACE*							
		rs4343		G	GG	AA	GA	
			Total	0.49	0.24	0.26	0.49	0.7060
			ACEI	0.48	0.24	0.27	0.48	0.4723
			Non-ACEI	0.51	0.25	0.22	0.52	0.5517
		rs4353		A	AA	GG	AG	
			Total	0.49	0.24	0.26	0.51	0.7505
	*ABO*							
		rs495828		T	GG	TT	GT	
			Total	0.23	0.61	0.07	0.32	0.0383
			ACEI	0.24	0.59	0.07	0.34	0.0488
			Non-ACEI	0.17	0.69	0.04	0.28	0.7152
		rs8176746		A	CC	AA	CA	
			Total	0.07	0.87	0.01	0.12	0.9490
			ACEI	0.06	0.88	0.01	0.11	0.9699
			Non-ACEI	0.08	0.84	0.01	0.15	0.9939

MAF: Minor allele frequency. HWE: Hardy-Weinberg equilibrium. *ACE*: ACE gene; *ABO*: ABO blood type gene; *AGTR1*: angiotensin II receptor type 1 gene; *BDKRB1*: bradykinin receptor B1 gene.

### Prognostic capacity of genetic score A

**Fig 2:**
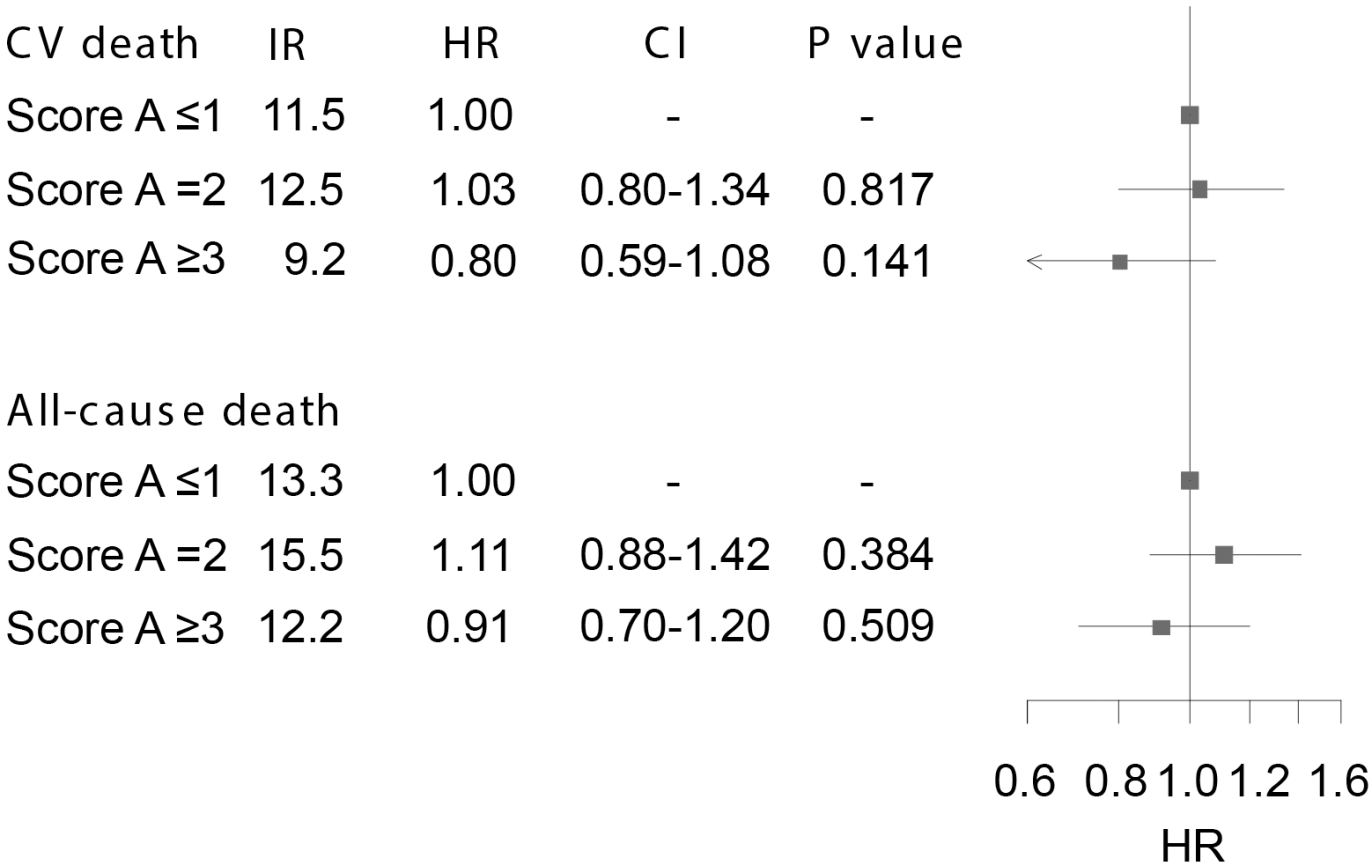
Risk of cardiovascular (CV) death and all-cause death in angiotensin-converting enzyme inhibitor-treated patients with congestive heart failure stratified by genetic score A. Results from adjusted multivariate Cox proportional hazard analyses with score A ≤1 used as reference. IR: Incidence rate per 100 patient-years; HR: Hazard ratio. CI: confidence interval. Error bars illustrate 95% CIs.

**Fig 3:**
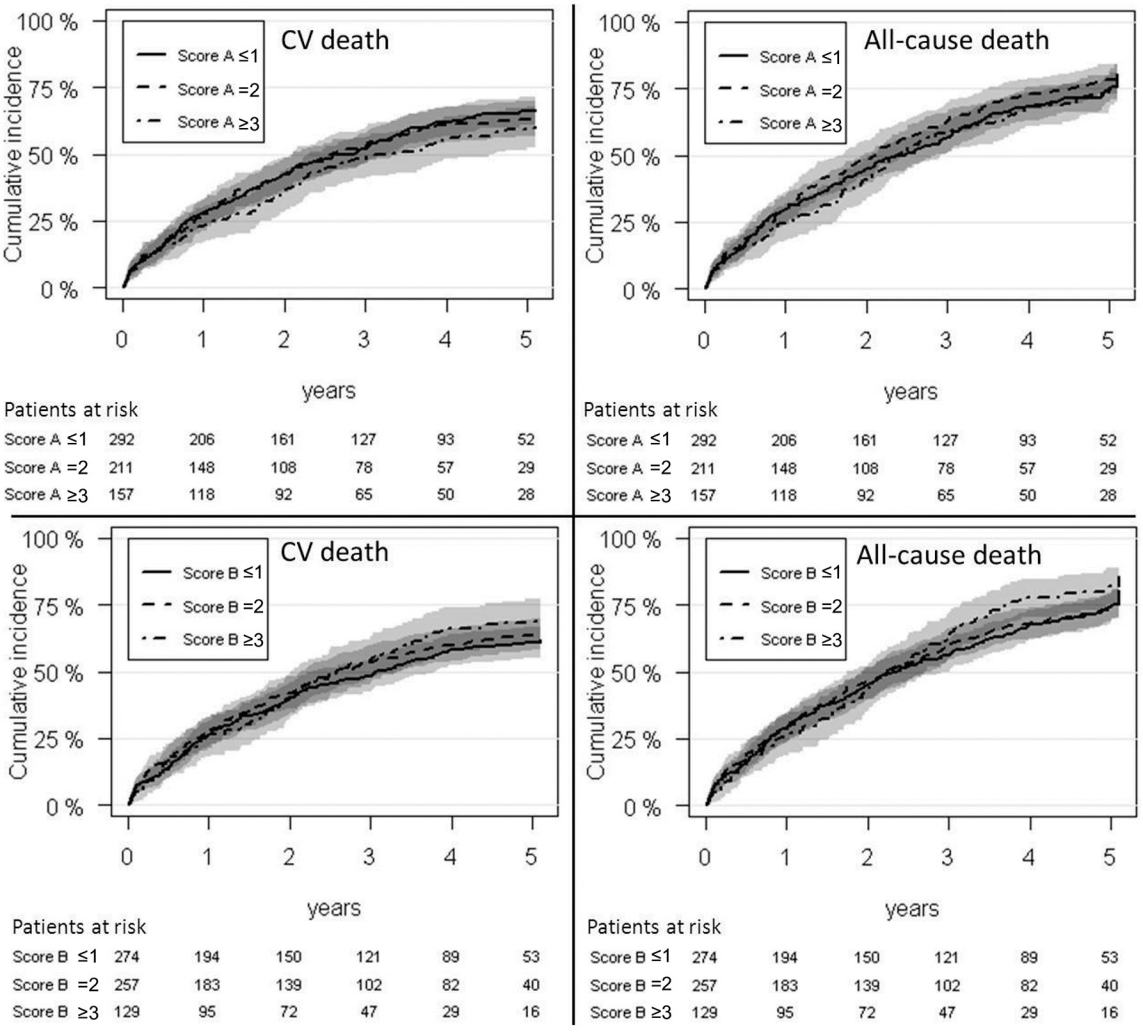
Cumulative incidence plots for cardiovascular (CV) death and all-cause death in angiotensin-converting enzyme inhibitor-treated patients with congestive heart failure according to genetic score A and B. The plots show the cumulative incidence estimates in % plotted against follow-up time in years. 95% confidence intervals are highlighted in gray scale nuances.

**Fig 4:**
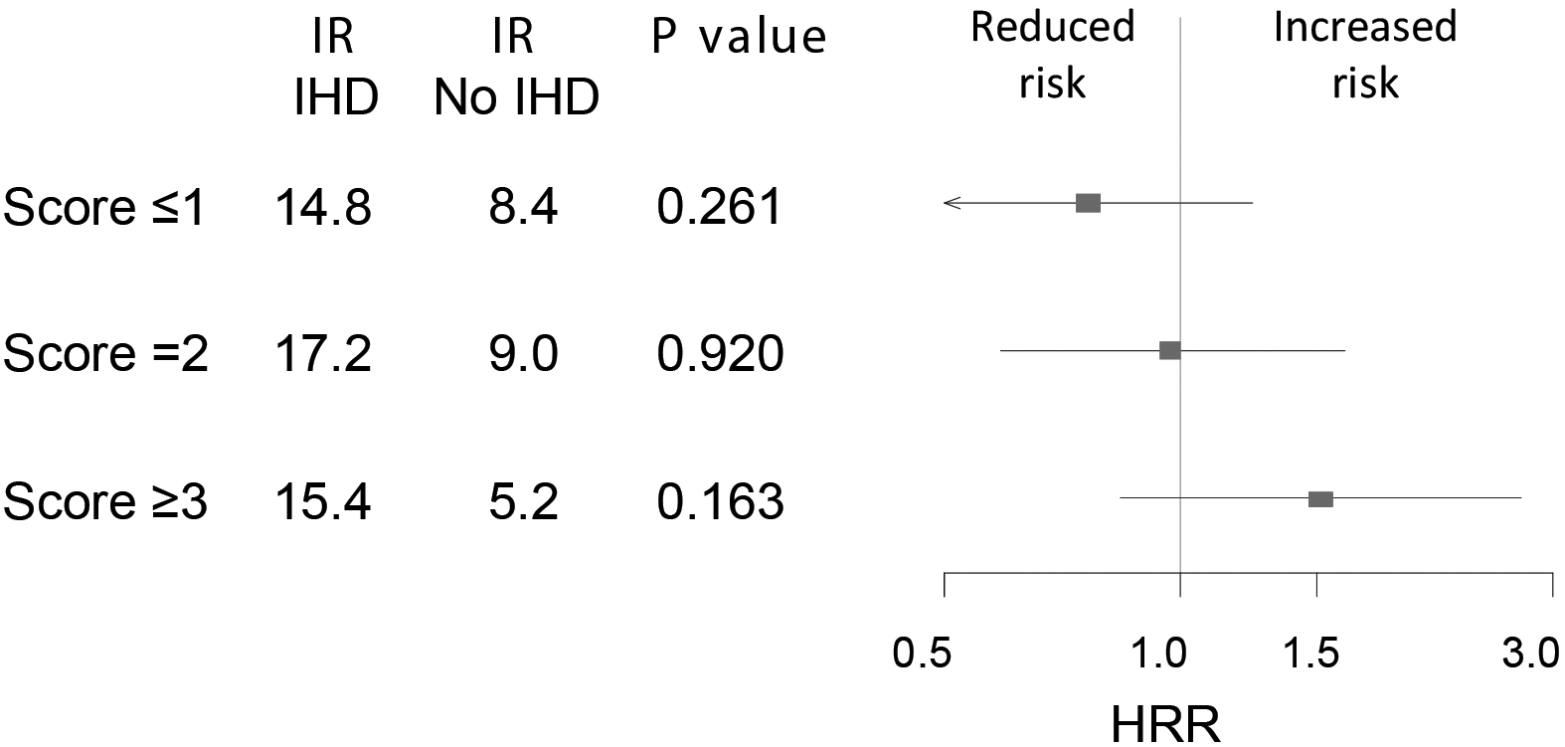
Difference in risk of cardiovascular death with different levels of genetic score A in angiotensin-converting enzyme inhibitor-treated patients with congestive heart failure (CHF) and ischemic heart disease (IHD) compared with no IHD. Results from adjusted multivariate Cox proportional hazard analyses. IR: Incidence rate per 100 patient-years; HRR: hazard rate ratio. Error bars illustrate 95% confidence intervals.

Crude IRs per 100 patient-years for both study endpoints are shown in Fig 2. The overall difference in cumulative incidence of CV death and all-cause death between patients with scores A ≤1, 2, and ≥3, was not significant (P = 0.346 and P = 0.486, respectively). Cumulative incidence plots for both endpoints are shown in Fig 3. There was no difference in risk of CV death between patients with score A ≤1 and 2, respectively (HR 1.03 [95% confidence interval (CI) 0.79–1.34], P = 0.817). However, compared with patients with score A ≤1, a non-significant nominal reduction in risk of CV death was found in patients with score A ≥3 (HR 0.80 [95% CI 0.59–1.08], P = 0.141) (Fig 2). HRs for all-cause death were 1.11 (95% CI 0.88–1.42, P = 0.384) and 0.91 (95% CI 0.70–1.20, P = 0.509), for subjects with score A = 2 and ≥3, respectively, compared to patients with score A ≤1 (Fig 2). Comparable results were found when endpoints were calculated for ACEI-treated patients after 5 years of follow-up and in additional analyses of patients not treated with ACEIs (Tables 4 and 5). Accordingly, no interaction was found between score A and ACEI treatment on CV death or all-cause death, respectively, when both ACEI-treated and ACEI-nontreated patients were included in the model (P = 0.789 and P = 0.858, respectively). In a subsequent analysis we tested the prognostic capacity of score A in ACEI-treated patients with ischemic and non-ischemic CHF, respectively, and found no change in risk of CV death amongst patients with ischemic CHF, with HRs of 1.05 (95% CI 0.74–1.49, P = 0.797) and 0.98 (95% CI 0.66–1.44, P = 0.917), for patients with score A = 2 and ≥3 compared to score A ≤1, respectively. However, there was a reduced risk of CV death with increasing score A in patients with non-ischemic CHF, with HRs of 0.89 (95% CI 0.57–1.37, P = 0.585) and 0.56 (95% CI 0.33–0.96, P = 0.034) for those with score A = 2 and ≥3 compared to score A ≤1. Accordingly, although there were overlapping CIs for hazard rate ratios, the difference in risk of CV death between ACEI-treated patients with ischemic versus non-ischemic CHF, showed a trend for increased risk of CV death with increasing score A in patients with ischemic CHF (P = 0.332 [Fig 4]). Finally, the prognostic value of score A did not change when analyses were restricted to only include patients treated with trandolapril, ramipril or perindopril (Table 6).

**Table 4:** Risk of cardiovascular (CV) death and all-cause death with genetic scores A and B in patients treated with angiotensin-converting enzyme inhibitors after 5 years of follow-up.

	HR	HR (CI)	HR (CI)	P value
	Score A ≤1	Score A = 2	Score A ≥3	
CV death	1.00	0.95 (0.73–1.24)	0.88 (0.65–1.19)	0.700
All-cause death	1.00	1.02 (0.80–1.30)	0.99 (0.76–1.30)	0.981
	Score B ≤1	Score B = 2	Score B ≥3	
CV death	1.00	1.03 (0.80–1.33)	1.09 (0.79–1.49)	0.876
All-cause death	1.00	0.97 (0.77–1.23)	1.08 (0.82–1.44)	0.757

HR: hazard ratio; CI: confidence interval.

**Table 5:** Risk of cardiovascular (CV) death and all-cause death with genetic scores A and B in patients without angiotensin-converting enzyme inhibitor treatment after 10 years of follow-up.

	HR	HR (CI)	HR (CI)	P value
	Score A ≤1	Score A = 2	Score A ≥3	
CV death	1.00	1.12 (0.68–1.84)	0.87 (0.50–1.53)	0.707
All-cause death	1.00	1.30 (0.82–2.05)	0.98 (0.58–1.65)	0.450
	Score B ≤1	Score B = 2	Score B ≥3	
CV death	1.00	0.89 (0.52–1.48)	0.70 (0.37–1.28)	0.495
All-cause death	1.00	0.88 (0.54–1.42)	0.74 (0.42–1.31)	0.590

HR: hazard ratio; CI: confidence interval.

**Table 6:** Risk of cardiovascular (CV) death with genetic scores A and B in patients treated with trandolapril, ramipril or perindopril.

	HR	HR (CI)	HR (CI)	P value
	Score A ≤1	Score A = 2	Score A ≥3	
Trandolapril	1.00	0.90 (0.61–1.35)	0.64 (0.39–1.05)	0.210
Ramipril	1.00	0.84 (0.42–1.66)	0.87 (0.43–1.74)	0.853
Perindopril	1.00	1.01 (0.48–2.14)	0.91 (0.40–2.07)	0.966
	Score B ≤1	Score B = 2	Score B ≥3	
Trandolapril	1.00	0.97 (0.65–1.47)	1.30 (70.73–2.30)	0.595
Ramipril	1.00	0.66 (0.32–1.39)	0.90 (0.41–1.97)	0.542
Perindopril	1.00	0.88 (0.40–1.90)	1.11 (0.47–2.61)	0.859

HR: hazard ratio; CI: confidence interval.

### Prognostic capacity of genetic score B

**Fig 5:**
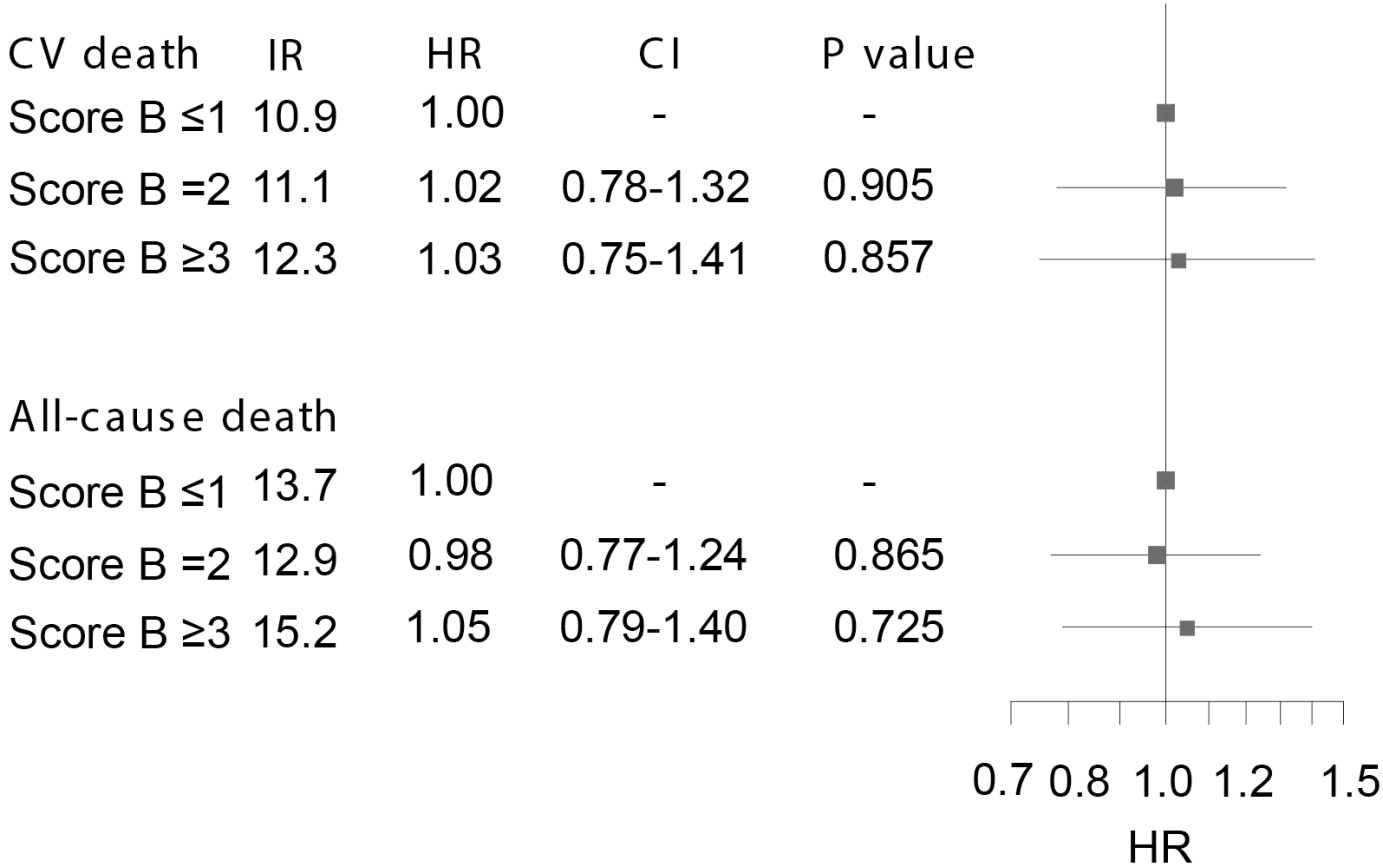
Risk of cardiovasclar (CV) death and all-cause death in angiotensin-converting enzyme inhibitor-treated patients with congestive heart failure stratified by genetic score B. Results from adjusted multivariate Cox proportional hazard analyses with score B ≤1 used as reference. IR: Incidence rate per 100 patient-years; HR: Hazard ratio. CI: confidence interval. Error bars illustrate 95% CIs.

Crude IRs per 100 patients-years for both study endpoints are shown in Fig 5. The overall difference in cumulative incidence of CV death and all-cause death between patients with scores B ≤1, 2, and ≥3, was not significant (P = 0.515 and P = 0.486, respectively). Cumulative incidence plots for both endpoints are shown in Fig 3. We found no difference in risk of CV death or all-cause death between subjects with score B ≤1, 2 or ≥3, respectively (Fig 5), and there was also no association between score B and either study endpoint after 5 years of follow-up (Table 4). In addition, score B had no significant influence on the prognosis in patients not treated with ACEIs (Table 5) and there was no interaction between score B and ACEI treatment on CV death or all-cause death when both ACEI-treated and ACEI-nontreated patients were included in the model (P = 0.799 and P = 0.699, respectively). Finally, the prognostic value of score B did not change when analyses were restricted to only include patients treated with trandolapril, ramipril or perindopril (Table 6).

## Discussion

In the present cohort study of ACEI-treated patients with CHF, we examined the prognostic value of two distinct pharmacogenetic scores A and B that have previously been found to influence ACEI efficacy and ACE activity in patients with stable IHD and hypertension, respectively. No association was found between these two scores and CV death and all-cause death during up to 10 years of follow-up. These findings were corroborated by sensitivity analyses, where endpoints were calculated after 5 years of follow-up and in analyses of patients not treated with ACEIs. However, subgroup analyses showed a trend for increased mortality with increasing score A in ACEI-treated patients with ischemic CHF compared with non-ischemic CHF. Our study results therefore suggest that score A and B do not predict mortality in ACEI-treated patients with CHF irrespective of etiology, but that score A may be of such value in patients with ischemic CHF.

The individual response to various drugs used for treatment of CHF is highly variable and this variability cannot solely be explained by differences in clinical characteristics of patients [19–21]. Accordingly, genetic variations have been suggested to have a considerable effect on drug response and in patients treated with ACEIs the *ACE* I/D polymorphism has been a focus of attention [9, 22, 23]. Indeed, high circulating ACE activity has been associated with this polymorphism and with worse cardiovascular outcomes in CHF patients [24]. Nonetheless, the utility of the *ACE* I/D polymorphism for pharmacogenetic risk stratification in CHF patients remains contentious, which may be explained, in part, by an anticipated small impact of this polymorphism on ACEI efficacy, and by the fact that published results have been limited by small study populations [22, 25, 26]. A few other genetic polymorphisms have separately been associated with the efficacy and safety of ACEIs, including some of those represented in the current study (rs4343, rs495828 and rs817646) [10, 27]. However, to our knowledge only one study, which examined the SNPs comprising score A of our study in patients with stable IHD, has hitherto used more than one SNP to provide an incremental pharmacogenetic risk score for prediction of survival in ACEI-treated patients [9, 11, 22].

In the present study of patients with CHF treated with or without ACEIs, we did not find any significant association between score A or B, and CV death and all-cause death, respectively. It is likely that the effect sizes of the examined SNPs in score A and B were too small to be detected in survival analyses. Also, the relatively small patient sample size of the study reduced its statistical power. Previously, the prognostic importance of score A was documented by a candidate gene (total of 12 genes) approach in patients with stable coronary artery disease that were approximately 10 years younger than our patient population and had a 5-year mortality <10%, while score B was derived from a genome-wide association study of patients of around 40 years of age with hypertension that examined effects on plasma ACE activity [10, 11]. Indeed, in agreement with the complex biological networks regulating the renin-angiotensin-aldosterone system (RAAS) activity and ACEI pharmacokinetics and pharmacodynamics in different cardiovascular disease states, ultimately only a polygenetic or genome-wide approach in large populations of CHF patients treated with ACEIs may provide more definitive answers. Of note, the genotype frequency of rs495828 of *ABO* in our study population was not in Hardy-Weinberg equilibrium, but this aberration was only of borderline significance. There was a higher degree of LD in *ACE* among the European HapMap individuals, where all investigated SNPs were highly correlated as compared to the HapMap Project population of Han Chinese subjects (Fig 1) [15]. Accordingly, we also found high correlation between the two tag SNPs rs4343 and rs4353 in the ECHOS population. Previous fine-mapping of *ACE* amongst Asian hypertensive patients identified a recombinant break point between rs4344 and rs4353 located adjacent to rs4343 that defined haplotypes with different ACE activities [12]. These different LD patterns of the examined SNPs between Europeans and Asians should be taken into account in the interpretation of the results of the current study and those reported in the literature [6–11].

Several large studies have shown that patients with ischemic CHF, independent of blood pressure reduction, benefit from ACEI treatment with a relative reduction in fatal outcomes of approximately 20% [19–21]. However, patients with ischemic CHF appear to have less improvement in symptoms, inferior hemodynamic response, and more limited amelioration of left ventricular dysfunction with contemporary anti-congestive treatment (including ACEIs) as compared to patients with non-ischemic CHF, and IHD has been shown to independently predict mortality in patients with CHF [28–30]. Moreover, ischemic CHF has been associated with more pronounced neuro-humoral activation and T-cell-mediated immune responses [31]. These differences between ischemic and non-ischemic CHF might reflect important variations in relative contributions of systemic and local effects of RAAS as well as differences in a range of other pathogenic and regulatory mechanisms [32, 33]. Accordingly, it is plausible that patients with ischemic CHF respond differently to ACEIs as compared to patients with non-ischemic CHF and these mechanisms may have contributed to our finding of increased risk of CV death with increasing score A in patients with ischemic CHF only. Also, ACEIs differ in their pharmacokinetic and pharmacodynamic profiles, e.g., with different lipophilicities and tissue penetration, which is likely to influence their efficacy, albeit that we found comparable results between the three most used ACEIs in our study, i.e., trandolapril, ramipril and perindopril, which differ in their lipophilicities [34–37]. Moreover, the relationship between systemic RAAS inhibition vs. RAAS inhibition at the tissue and intracellular level in respective pathophysiological target zones, e.g., arterial wall, heart, and kidney, respectively, is less clear at present [33, 38]. Interestingly, reports have documented the existence of cross-talk between the adrenergic system and RAAS, and treatment with beta-blockers and ACEIs may act through shared pharmacodynamics pathways [39–41]. In this regard, a recently published pharmacogenetic study that was also conducted in the ECHOS population, showed increased risk of death amongst carvedilol-treated patients with the combination of gain-of-function SNPs in the adrenergic β-1 receptor gene (Arg-389-homozygous) and angiotensinogen gene (Thr174-homozygous), and a loss-of-function SNP in the adrenergic β-2 receptor gene (Gln27-carrier), respectively [42]. These findings clearly highlight the necessity to include genetic variants from several relevant pathophysiologic pathways in pharmacogenetic investigations of complex diseases such as CHF.

### Limitations

In addition to the considerations mentioned above, other limitations apply to the interpretation of the present work. For example, although the current study was based on a cohort of patients with comparable baseline characteristics who were recruited for a randomized controlled trial, the risk of unmeasured confounding is inherent to retrospective cohort studies. Also, as opposed to score A, score B was originally associated with systemic ACE activity and blood pressure response to ACEIs, but not directly with clinical outcomes in patients receiving ACEIs [10, 11]. In addition, our study was limited by a relative small sample size and we did not differentiate between different ACEIs (which can have different pharmacokinetic and pharmacodynamic characteristics) or ACEI doses [34–36]. We did not measure plasma angiotensin II levels before and after ACEI treatment and were therefore not able to assess the level of systemic ACE inhibition that was achieved. It should also be mentioned that the statistical analyses were based on baseline characteristics of the study population and we did not correct for possible changes in ACEI treatment over time, however, the proportion of patients with CHF initiated with ACEIs who still redeem their ACEI prescriptions after five years has in a previous Danish registry study been found to be 79% [43].

### Conclusion

The current study of patients with CHF treated with ACEIs did not find any significant association between CV death and all-cause death and two genetic scores (score A and B) that have previously been associated with ACEI efficacy. However, the analyses suggested a trend for increased risk of CV death with increased score A in ACEI-treated patients with ischemic CHF relative to non-ischemic CHF. While this finding warrants further study, genome-wide association studies and analyses that incorporate multiple gene interactions may be necessary to provide more detailed insights into the potential role of pharmacogenetic risk stratification of patients with CHF.
